# Evaluation of the Histological Changes in the Structure of the Minor Salivary Glands in Patients With Oral Submucous Fibrosis (OSMF)

**DOI:** 10.7759/cureus.31576

**Published:** 2022-11-16

**Authors:** Mimansha Patel, Nishath Sayed Abdul, Dushyantsinh Vala, Mahesh Shenoy, Vinod Birra, Jyoti Wasti, Ramanpal Singh

**Affiliations:** 1 Department of Oral Pathology and Microbiology, Triveni Institute of Dental Sciences, Hospital and Research Centre, Bilaspur, IND; 2 Department of Oral Pathology, College of Dentistry, Oral Diagnostic Sciences, Riyadh Elm University, Riyadh, SAU; 3 Department of Oral and Maxillofacial Pathology and Oral Microbiology, Government Dental College and Hospital, Jamnagar, IND; 4 Department of Dental Surgery, Andhra Medical College, Visakhapatnam, IND; 5 Department of Periodontics, Government Dental College, Raipur, IND; 6 Department of Oral Medicine and Radiology, New Horizon Dental College and Research Institute, Chhattisgarh, IND

**Keywords:** oropharynx, incisional biopsy, histological changes, osmf, minor salivary gland

## Abstract

Background: The minor salivary glands (MSGs) are critical components of the mouth's delicate environment. The pre-malignant changes of oral submucous fibrosis (OSMF) have been associated with a decline in the quality of life and an uptick in the prevalence of oral malignancies.

Aim: The aim was to provide evidence of the histological alterations in minor salivary gland structure seen in individuals with OSMF.

Methods and materials: A total of 106 confirmed cases of OSMF were enrolled in the study. In order to perform an incisional biopsy, we first collected the patient's complete demographic and clinical history. Using a Vernier calliper, the inter-incisal distance was used to evaluate^ ^the mouth opening of the patients. An incisional biopsy of the buccal mucosa was carried out using a 6 mm diameter punch and local anaesthesia. After the appropriate demographic and medical information had been gathered. Acinar cells and the surrounding stroma of tissue slices were observed under a light microscope for alterations. The cytoplasm, nucleus morphology, cellular shape, mucin pooling, and acinar outline of acinar cells were all examined by the researchers. It was taken into account to classify OSMF histologically based on variations in juxta epithelial hyalinization.

Results: Multiple aetiologies for the symptoms of OSMF were reflected in the patient's histological abnormalities in the minor salivary glands. On the measurement of the diameter of acini, we discover that the average area of salivary gland acini in OSMF patients is smaller than in the normal group indicative of a decrease in size. The number of functional acini in OSMF is fewer than compared in the control group.

Conclusion: Because of the findings of this study, we now have a better understanding of the factors that play a role in the incidence of dryness of the oral and pharyngeal mucosa (OSMF), although it has to be mentioned that no major impact of OSMF on minor salivary glands was observed in our study.

## Introduction

The minor salivary glands (MSGs) are an essential component of the oropharynx and are crucial for preserving oral cavity equilibrium. They range in number from 400 to 500 and are present in almost every part of the oropharynx, with the exception of the gingiva and the anterior portion of the hard palate. The buccal and labial MSGs are found near the stratified squamous epithelium, according to histological study, whereas the remaining MSGs are found in the submucosal and mucosal layers of the oral mucosa. If the stromal and submucosal layers of the oral mucosa have been damaged, then MSGs are very likely present [1,2].

Oral submucous fibrosis (OSMF), a potentially malignant disorder, is associated with a higher risk of tumour growth and poor oral health-related quality of life. Oral mucosa hyalinization and the build-up of thick strands of collagen are two hallmarks of this condition. Hypoplastic oral mucosa, rete ridge atrophy, and juxta epithelium inflammatory process reaction are further distinguishing characteristics. OSMF has seen a significant change in terms of how the condition is graded. The many grading systems that have been presented so far include both diagnostic and histopathologic characteristics [3,4]. The amount of deposition of collagen in the mucosa as well as submucosa as well as around the blood vessels has been given great consideration, particularly in histopathological criteria. However, MSGs, a very important component of the oral mucosa, have not received enough attention. Therefore, this study was done to show how the structure of the MSGs changes histologically in people with OSMF and to answer whether it really has a significant/noticeable impact on the minor salivary glands of its sufferers, which might lead to breakthroughs in its swift diagnosis.

## Materials and methods

One hundred and six OSMF cases were enrolled in our study. For the diagnosis, Pindborg and Sirsat's [5] clinical criteria were applied. The ethical clearance was taken from Andhra Medical College with reference number 2020/126. At the time of the incisional biopsy, the patient's complete demographic and clinical history had been collected. Using a Vernier calliper, we measured the distance between the two incisors to calculate the jaw's opening in millimetres. After gathering all the socio-demographic and medical information, a 6 mm diameter punch was used to conduct an incisional biopsy of the buccal mucosa under local anaesthesia.

The tissue specimens were stained with Van Gieson and Alcian Blue stain and Mucicarmine stain. Tissue sections from 10 previously documented instances of OSMF with signs of xerostomia were collected from the archives of the Department of Oral Pathology and stained with hematoxylin and eosin. In order to assess acinar and stromal alterations, tissue slices were subjected to light microscopy. Acinar alterations included alterations in the cytoplasm, altered nuclear morphology, acinar contour, cellular outline, and mucin pooling. Changes in the stroma were detected, which included the appearance of new blood vessels, ductal alterations, and immunological infiltration. Pindborg and Sirsat's histological classification of OSMF [5] based on changes in juxta epithelial hyalinization was taken into account.

Clinical oral dryness score (CODS)

The CODS, which is centred on a scale from 1 to 10, with each inquiry indicating the moisture loss in the oral cavity, was utilised to produce a quantification for dryness. This questionnaire has a solid scientific foundation after undergoing all the required validation methods. There are 10 clearly defined questions, and each one receives a score of 1. To establish a total score for quantitative tests, all the ratings were combined together. A high overall score suggests that mouth dryness is more severe. The examiner gave each score a value based on what he or she saw in the mouth of the patient, giving a final score between 0 and 10.

Calculation of salivary secretion

To measure salivary flow, the weighing approach was used, and an electronic digital balance was employed. If we assume that 1 gramme is equivalent to 1 millilitre of saliva, then we can calculate the data by dividing the weight change of the vial collected before and after sample collection by the time we spent on the task. Saliva samples were taken from each person at once. Given that the band's surface area was 3.5 cm2, the saliva flow rate was calculated by dividing the production rate by 3.5. The measurement is in microliters per square centimetre per minute (L/cm2/min), which is the unit of measurement.

Grading of MSG histopathological changes with OSMF

The buccal mucosa was the location of the biopsy in each case, so buccal MSGs were the focus of the entire analysis. Forty of the instances had fibrosis inside the gland and surrounding the salivary gland acini, and we observed peri-glandular fibrosis around all of the MSGs (intra-glandular). Additionally, the severity of the fibrosis around the MSGs varied, and we classified it as mild, moderate, and severe. There was also grading as shown in Table 1.

**Table 1:** Grading for the involvement of minor salivary gland in oral submucous fibrosis

Grading	Histopathology parameters	Number
1	Peri-glandular + mild fibrosis	30
2	Peri-glandular + moderate fibrosis/intra-glandular + mild fibrosis	48
3	Peri-glandular + severe fibrosis/intra-glandular + moderate to severe fibrosis	28

The CODS data, mouth-opening data, salivary flow rate data, and other segment data were summarised using the mean and standard deviation as part of the statistical analysis of our investigation. The average CODS, mouth opening, and salivary flow rate of those exposed to various levels of MSG were compared using a one-way analysis of variance and Tukey's post hoc test (post hoc correlations). A test performed using SPSS Statistics for Windows, Version 17.0 (Released 2008; SPSS Inc., Chicago, United States) was considered statistically significant if the p-value was less than 0.05.

## Results

One hundred and six participants in the current study had been clinically and histopathologically determined to have OSMF. Patients with OSMF had an average age of 32.73±12.42 years. The OSMF group consisted of 92 men and 14 women. This was consistent with the fact that Indian men tend to chew betel quid more frequently [6]. With a range of 0 mm to 37 mm, the average mouth opening for OSMF patients was 22.17 mm. In comparison to the control group, OSMF had less functioning acini.

Grading of MSG with OSMF histological changes

Fibrosis was primarily seen across the acini and around the MSGs (54 occurrences) on the histological investigation (52 cases). Last but not least, out of 106 OSMF patients, 30 individuals met the requirements (Table 1) for grade I, 48 individuals met the criteria for grade II, and 28 individuals met the criteria for grade III. Using OSMF, we compared the MSG participation grade with mouth opening. The maximum mouth opening was recorded in grade one (33±4.98), followed by grade two (21.96±5.42), and grade three (12.53±6.32) with statistically significant differences between them. When the mouth openings of the three groups were compared, there were statistically significant differences between the groups.

Clinical oral dryness score and grading of MSG involvement with OSMF

Compared to grade II (5.15±0.73) and grade I (3.68±0.96), the CODS was higher in grade III (6.6±2.10). There was a trend toward reduced CODS levels at increased levels of MSG participation, and this trend was statistically significant. In addition, there was a statistically significant difference in CODS between the three levels of grading.

Rate of MSG flow and MSG's role in OSMF grading

A correlation was found between the histopathological grading of MSG interaction with OSMF and the buccal MSG flow rate. When MSG was applied, the flow rate of Grade I was greater than that of Grades II (5.97±2.10) and III (4.5±0.87). In terms of MSG flow rate, there were statistically significant variations between grades I and II (p=00002*) and grades I and III (p=00002). MSG involvement in grades I and III fibrosis, on the other hand, did not differ statistically significantly (p =0.74290) (Table 2).

**Table 2:** Comparison of mouth opening, clinical oral dryness score, and salivary flow rate in various minor salivary gland involvement grades MSG- minor salivary gland, SD- standard deviation, n- number

MSG Involvement Grade	n	Mean SD	F ratio	P value
Mouth Opening				
I	30	33 ± 4.98	79.949	I versus II: < .00002
II	48	21.96 ± 5.42		I versus III: < .00002
III	28	12.53 ± 6.32		II versus III: < .00002
Clinical Oral Dryness Score				
I	30	3.46 ± 0.96	52.97	I versus II: p < .00002
II	48	5.15 ± 0.73		I versus III: p = .00002
III	28	6.6 ± 2.10		II versus III: p < .00002
Salivary Flow Rate				
I	30	9.13 ± 3.10	31.49	I versus II: < .00002
II	48	5.97 ± 2.10		I versus III: < .00002
III	28	4.4 ± 0.87		II versus III: p = .74290

## Discussion

During the examination of patients with OSMF, we measured the diameter of their salivary gland acini. It was discovered that the average area of salivary gland acini in OSMF patients was smaller compared to the normal group indicative of a decrease in size. The most significant finding observed during staining was under Alcian blue at Ph-3.2. The dye known as Alcian Blue is designated copper phthalocyanine. It has positive charge groups that may form linkages with certain polyanions. Sulfated and carboxyl radicals of acid mucin stain positively, but phosphate radicals of nucleic acid do not respond to such reactions. Changes in the solution's pH are indicative of the acid mucin species’ presence. Patients with OSMF in our study had less consistent staining in the minor salivary gland, indicating a decrease in the number of functional acini. Hyalinization of the oral mucosa and the development of dense collagen fibres are hallmarks of this condition. A hypoplastic oral mucosa, rete ridge atrophy, and a juxta epithelium inflammatory response are three more differentiating markers of this condition [7,8]. In terms of how the condition is rated, OSMF has undergone significant alteration. Both diagnostic and histopathologic traits are taken into account by the numerous grading schemes that have been established thus far. Particularly in histological criteria, the amount of collagen deposition in the mucosa, submucosa, and surrounding blood vessels has been carefully considered. MSGs are a very significant component of the oral mucosa, but not nearly enough attention has been paid to them [9,10]. In our study patients were observed in the age group of around 30 years who happen to be categorized as young adults. According to Pindborg and Sirsat’s study [5], the age range of 40-49 years old had the highest prevalence of OSMF patients. This shifting pattern of the illness now suggests that a greater number of people in their younger years are affected by it. This may be the result of increasing social contacts as well as the enhanced economic freedom they experience at this age in a quickly rising country like India. Because of this, when they are this age, they engage in a variety of chewing habits such as betel nut, gutka, paan masala, smoking, and drinking, amongst other things, either to alleviate tension, as a fashion, or due to the pressure of their peers [11].

To keep the oral cavity in check, the MSGs, which are located in the oropharynx, are essential. In terms of MSG flow rate, there were statistically significant variations between grades I and II and grades I and III. MSG involvement in grades I and III fibrosis, on the other hand, did not differ statistically significantly, which was in accordance with a study done by Ingle et al. [12]; a comparison was made between the MSG flow rate and a suggested grading system for the MSG's cooperation with OSMF. As the degree of MSG engagement with fibrosis grew, there was a noticeable tendency toward a decreased flow rate. The gingiva and the anterior portion of the hard palate are the only areas where MSG’s not found in. In terms of oral mucosal histology, they are found in both the mucosal and submucosal layers. [10,11]. MSGs are located in close proximity to the squamous stratified epithelium in the buccal and labial mucosa. Because of this, it is safe to assume that MSGs are always present in any disorder that affects the oral mucosa's stromal area and sub-mucosa. Each biopsy was taken from the buccal mucosa, and buccal MSGs were the main subject of the analysis. In 40 cases, there was fibrosis both inside the gland and outside the acini, but in all of the cases, there was peri-glandular fibrosis around the MSGs (intra-glandular). We also told the difference between mild, moderate, and severe fibrosis around the MSGs based on how bad it was [13-16]. When the mouth openings of the three groups were compared, there were statistically significant differences between the groups which were not in accordance with the study conducted by Rooban et al [17] who came to the conclusion that the link between the clinical staging of mouth opening and histological grading of fibrosis did not demonstrate any statistical significance. The reason for the differences in findings was that the staging method that was employed in research by Khanna and Andrade (1995) [18] was unique in comparison to the staging systems used in other investigations. In the research carried out by Ingle et al. [12], it was shown that the CODS value rose when there was a higher level of MSG engagement with OSMF. This was one of the findings of the research. According to these data, MSG fibrosis may have been the source of the decrease in the functioning of the glandular secretory cells, which ultimately led to a drop in salivary output. However, the participation of buccal MSG will not, by itself, significantly contribute to dryness in the mouth cavity; this result was not in accordance with our present study, which showed that there was a trend toward reduced CODS levels at increased levels of MSG participation, and this trend was statistically significant. In addition, there was a statistically significant difference in CODS between the three levels of grading.

The correlation between xerostomia and OSMF observed in our study has been reported in studies by Sarode et al. [19] and Rao et al. [20]. In their routine histopathology practice, Sarode et al. [19] observed fibrosis surrounding minor salivary glands, distended acini, obliteration of acinar lumen, and loss of interstitial spaces. Based on those features, they hypothesized that fibrosis in OSMF drove localized peripheral autonomous neuropathy in minor salivary glands, which led to dysfunctional myoepithelial cells. Those dysfunctional myoepithelial cells were unable to contract and expel saliva out of the salivary secretary unit, which thus led to xerostomia in the patients selected for their investigations. Rao et al. [20], in their investigation, reported the incidence of xerostomia during the intra-oral examination of patients in the moderate and advanced stages of OSMF in conjunction with other signs such as stomatitis, burning sensation, loss of taste sensation, a gradual decrease in mouth opening, difficulty in whistling, vesicle formation, petechiae, rigid oral mucosa, difficulty in blowing the cheeks, defective gustatory sensation, and blanching of the oral mucosa (especially of the soft palate), buccal mucosa, labial mucosa, tongue, and floor of the mouth, similar to what we observed in our study.

The current study has some limitations. Though fibrosis provides a reasonable indicator of the physical functioning of the MSG, this does not specify the full degree of degradation taking place at the cellular scale. Only the analysis of molecular markers linked to degeneration can be used to quantify this. The second drawback was that only buccal mucosa MSGs were taken into account because this was the most typical biopsy location in OMSF patients. This did not accurately reflect the whole state of all MSG functional impairments. The fact that it is almost impossible to examine all MSGs histologically at the level of incisional biopsy is also true, though. Also, despite the two studies mentioned, the link between the incidence of xerostomia in OSMF patients is still not conclusively established. This warrants the need for further investigation into this domain.

## Conclusions

To the best of our knowledge, there is no scientific evidence that elucidates the impact of OSMF on either the main or the small salivary glands found in humans. In the course of our research, we saw that the acini were degenerating, as shown by the loss of cellular and acinar contour, as well as alterations in the nucleus and mucin pooling. It's possible that these are the reasons why OSMF patients have been observed to suffer from xerostomia in some stages of the disease as mentioned in our discussion. Imaging and examination of the histological abnormalities in the major salivary glands of these individuals may assist further to understand the aetiology of the occurrence of xerostomia in oral submucous fibrosis.

In addition to being unable to carry out their typical activities, patients who have OSMF have tiny salivary glands that are visibly altered physically. Patients who have OSMF also have difficulty swallowing. Because OSMF has an effect on the patient's olfactory system, this is the result. Because of the findings of this study, we now have a better understanding of the factors that play a role in the development of dryness of the oral and pharyngeal mucosa (OSMF).
